# The Need for Next-Generation Antivenom for Snakebite Envenomation in India

**DOI:** 10.3390/toxins15080510

**Published:** 2023-08-18

**Authors:** Muralidharan Vanuopadath, Karthika Rajan, Aswathy Alangode, Sudarslal Sadasivan Nair, Bipin Gopalakrishnan Nair

**Affiliations:** School of Biotechnology, Amrita Vishwa Vidyapeetham, Kollam 690 525, Kerala, India; muralidharanv@am.amrita.edu (M.V.); karthikarajan@am.amrita.edu (K.R.); aswathya@am.amrita.edu (A.A.); sudarslal@am.amrita.edu (S.S.N.)

**Keywords:** snake venomics, antivenomics, immunological cross-reactivity, Indian polyvalent antivenom, next-generation antivenom, aptamers, phage display, natural products, small-molecule inhibitors

## Abstract

The limitations posed by currently available antivenoms have emphasized the need for alternative treatments to counteract snakebite envenomation. Even though exact epidemiological data are lacking, reports have indicated that most global snakebite deaths are reported in India. Among the many problems associated with snakebite envenomation, issues related to the availability of safer and more efficient antivenoms are of primary concern. Since India has the highest number of global snakebite deaths, efforts should be made to reduce the burden associated with snakebite envenoming. Alternative methods, including aptamers, camel antivenoms, phage display techniques for generating high-affinity antibodies and antibody fragments, small-molecule inhibitors, and natural products, are currently being investigated for their effectiveness. These alternative methods have shown promise in vitro, but their in vivo effectiveness should also be evaluated. In this review, the issues associated with Indian polyvalent antivenoms in neutralizing venom components from geographically distant species are discussed in detail. In a nutshell, this review gives an overview of the current drawbacks of using animal-derived antivenoms and several alternative strategies that are currently being widely explored.

## 1. Introduction

Snakebite envenoming (SBE) is a significant threat to the people living in the world’s tropical regions [1]. Globally, 5.4 million snakebites are reported yearly, resulting in 1.8 to 2.7 million envenomation cases, 8100 to 138,000 deaths, and three times as many permanent disabilities and amputations [2]. A recent study indicated that approximately 64,000 deaths are reported yearly due to snakebites in India, accounting for more than 50% of the global deaths reported due to snakebites [3]. Hence, due to the degree of severity, lack of proper treatment, increasing mortality rates, and high treatment costs, in June 2017, the World Health Organization included snakebite envenoming under the neglected tropical disease category [4]. By doing so, the WHO aims to achieve a 50% reduction in morbidity and mortality rates associated with snakebite envenomation by 2030. Several strategic plans were outlined by the WHO’s expert committee on snakebite envenomation. Some of these plans are based on control and prevention, improving ecological and epidemiological data, providing better treatment for envenomed patients, and addressing antivenom issues [2]. The ‘big four’ venomous snakes that are responsible for most snakebites and associated morbidity and mortality cases in India include *Bungarus caeruleus* (Common krait), *Daboia russelii* (Russell’s viper), *Echis carinatus* (Indian saw-scaled viper), and *Naja naja* (Indian cobra) [3]. Table 1 shows India’s medically important snake species and the corresponding availability of antivenoms used to treat SBE caused by these snakes.

## 2. Variation in Snake Venom Composition and Antivenom Efficacy

Snake venom is a complex cocktail of several inorganic and organic constituents, including carbohydrates, lipids, proteins, and metal ions. The major class of protein families present in snake venoms are distributed among the four families are as follows: phospholipase A_2_ (PLA_2_s), snake venom metalloproteases (SVMPs), snake venom serine proteases (SVSPs), and 3- finger toxins (3-FTxs) [6]. These proteins are commonly found across snake species but in varying proportion depending on the age, sex, region, and diet of the snake species [7]. Various clinical manifestations in snakebite patients are due to the action of these toxins. Targeting these proteins and neutralizing them will serve as a better treatment option for venom-associated pathologies. The pharmacological effects induced after a snakebite may result from the synergistic effects of all these components [6,8]. Though the snake venom proteome of the most medically important snake species has been studied, a detailed investigation of the influence of various factors such as diet, body size, gender, and geographical and habitat location has not been conducted [9,10,11,12]. Investigating and compiling all of this information might be useful in delineating the venom proteome and the impact of these in influencing the efficacy of Indian polyvalent antivenoms. Due to profitability issues, most companies stopped the production of antivenoms, which has severely affected the availability of antivenoms [13]. Hence, the only option is to optimize the existing antivenoms to treat snakebite envenomation [14,15,16,17]. A conventional treatment strategy available for snakebites is the intravenous administration of antivenom [18]. Antivenom production involves injecting snake venom into an animal host, usually a horse, which generates antibodies against the venom [19,20]. Subsequently, these antibodies are isolated from the animal and used as antidotes against snakebites. Although these are effective in neutralizing systemic toxins, the effective neutralization of local effects is limited [21,22,23], leading to morbidities associated with envenomation. This could partially be due to the onset of inflammatory mediator release immediately following the snakebite [24]. Snake venom toxins have antigenic determinants shared among phylogenetically distant snake species. Assessing the cross-reactivity of snake venom to polyvalent antivenoms is needed to understand the effectiveness of existing antivenoms. These antivenoms can also be used in testing venoms that are not included in the existing immunization mixture, which could eventually shed light on the clinical application and importance of antivenom specificity [15,16,25]. Paraspecific neutralization or the cross-neutralization of antivenoms is often seen in closely related venomous snake species that share homologous venom antigenic epitopes, even if they are from different geographical locations [26,27,28,29,30]. This might be helpful in the reduced usage of venom from more snakes for generating antivenom. Nevertheless, these must be tested in suitable animal models and validated clinically before being made commercially available. In vivo and in vitro preclinical studies can be performed to determine the effectiveness of a new antivenom formulation. In addition, the manufacturers need regulatory approval to generate pan-India or region-specific antivenoms. In all of these scenarios, the antivenom manufacturers must perform sufficient preclinical studies using a reference venom mixture of medically important snake species from a specific country or geographical location [13]. This helps to evaluate the efficacy of antivenoms, which is essential because reports show that venom variation plays a crucial role in determining the clinical efficacy of antivenoms [31,32,33,34]. As seen in Table 2, there are eight antivenom manufacturing companies in India. However, it is worth noting that three are not included in the WHO Snakebite Information and Data Platform [5]. Table 3 shows numerous reports on checking the cross-neutralization and preclinical efficacy of various Indian polyvalent antivenoms using venom from Indian snakes. As indicated earlier, though three of India’s antivenom manufacturers are not listed in the WHO’s database, many in vitro preclinical studies have been performed using their antivenoms (Table 3). 

## 3. Preclinical Studies of ‘Big Four’ Indian Snakes Using Indian Polyvalent Antivenoms

The neutralization efficacies of the antivenoms manufactured by different vendors are tested using in vivo animal models before they enter the market. However, as recommended by the WHO, in vitro preclinical studies need to be performed to evaluate the neutralization and binding capacities of these antivenoms with respect to individual snake species [13]. Interestingly, most studies use the venom from the big four snakes from various geographical locations to check various antivenom immunological responses. Out of the big four snakes, Indian cobra venom and Russell’s viper venom (Table 3) obtained from different geographical locations have been extensively studied. Venom proteome profiling of all these species indicated that the abundance and distribution of snake venom protein families vary across different geographical locations. It was also interesting to see that venom proteome variation also affects antivenom efficacy (Table 3). For example, though different groups have studied *Naja naja* from south India, the antivenom studies using the same venom indicated that their cross-neutralization potential varies [34,35,36,37]. This shed light on the need to generate antivenom from a geographical perspective. Nevertheless, several other factors, such as antivenom production and purification strategies, might also contribute to antivenom’s clinical effectiveness. Additionally, though eight antivenoms are commercially available (Table 2), none of the published reports have checked the preclinical evaluation of all these antivenoms in a single study. Also, it is interesting to note that most studies use antivenom from Bharat Serums, Premium serums & vaccines, and VINS Bioproducts. 

## 4. Lethal Envenoming by ‘Non-Big Four’ Snakes in India and the Antivenom Concern

Indian polyvalent antivenoms are generated by immunizing horses using the venom pooled from the ‘big four’ snakes collected by Irula snake catchers [38]. Most mortality cases are reportedly due to bites from ‘big four’ Indian snakes, and polyvalent antivenom is provided at the appropriate doses [38,39]. However, numerous in vitro and in vivo reports have substantiated the claim that the polyvalent antivenom often fails to recognize and bind to antigenic epitopes present even in the ‘big four’ species [37,39,40,41,42]. Apart from these, there are several other medically relevant species in India, including different species of kraits, cobras, pit vipers, saw-scaled vipers, and sea snakes [43]. Clinical reports also indicate that the number of envenomation cases and the complications from envenoming induced by these ‘non-big four’ snakes are also alarmingly increasing [44,45,46]. Clinical studies have shown that venom-induced consumption coagulopathy induced by *Echis carinatus sochureki* venom was not neutralized after providing Indian polyvalent antivenoms [47]. However, it is worth noting that specific antivenoms are unavailable for all of these snakes (Table 1). Reports have even indicated that Indian polyvalent antivenoms are ineffective in neutralizing the venom proteins present in some of these species [48]. Due to these concerns, the WHO recommends identifying and characterizing the venom proteome of medically important snake species from a particular geographical region and generating region-specific antivenoms after performing WHO-approved preclinical tests to check the neutralization efficacy of the generated antivenoms [13]. Table 3 shows that the antivenom neutralization studies are limited to the ‘big four’ snake species and that very few studies have considered the ‘non-big four’ snake species. In this regard, in addition to exploring venom proteome profiling, it is equally important to assess an antivenom’s immunological cross-reactivity towards all of the medically important snakes. 

**Table 3:** Antivenom neutralization studies using Indian polyvalent antivenom and snake venom from different geographical locations in India.

SI No.	Snake Species	Geographical Location of the Snake (s) Used for the Study	Antivenom (s) Used	Antivenom Neutralization Studies Performed	Observations/Inferences	Ref.
1	*Naja naja*	Northwestern (Rajasthan, Gujarat)	VINS	IAC	Antivenom efficacy varies according to the geographical location of the snake species.	[34]
2	*Naja naja*	Eastern India—(Burdwan District, West Bengal) Calcutta snake park, Kolkata	BSAV, PSAV	ELISA, WB, IAC	Both antivenoms showed poor immunological cross-reactivity to the low-molecular-mass proteins (<20 kDa) present in *Naja naja* venom.	[35]
3	*Naja naja*	Southern India—Tamil Nadu-Irula snake catcher’s society	BSAV, PSAV, VINS, and Virchow	ELISA, WB	The low molecular mass (<15 kDa) proteins showed poor immuno-recognition compared to the high- and mid-molecular-mass proteins.	[36]
4	*Naja naja*	Western India—Haffkine Institute, Mumbai	BSAV, PSAV, and Virchow	ELISA, WB, IAC, neutralization of enzyme activities and anti-coagulant activity	Poor recognition of proteins with low-molecular-mass (<20 kDa) toxins present in the cobra venom from Western parts of India.	[37]
5	*Naja naja*	Maharashtra (West India)	BSAV, PSAV, VINS, and Haffkine	ELISA, WB, LD_50_, ED_50_	All antivenoms recognized the venom antigenic epitopes in the ‘big four’ snake species more efficiently than other snake species.	[48]
6	*Naja naja*	India (exact location not mentioned)	VINS, BSAV	LD_50_,ED_50_	BSAV antivenom was very weak in recognizing venom from other krait and cobra species. However, the VPAV effectively neutralized venom from all Southeast Asian cobras, *B. candidus*, *N. naja*, and *Ophiophagus hannah* with varying potencies.	[49]
7	*Naja naja*	Western Ghats of India-Kerala	VINS, PSAV, Virchow	ELISA, WB	Antivenomics performed using VINS antivenom indicated that it detects and binds to low-molecular-mass proteins less effectively.	[50]
8	*Naja naja*	North—Punjab	BSAV, PSAV, Haffkine, and VINS	ELSA, WB, LD_50_, ED_50_	This study showed that all the antivenoms failed to neutralize *Naja naja* venom from desert populations. This study reiterated the need for the development of a pan-India antivenom that is effective against all snake species.	[51]
		South—(Tamil Nadu)				
		Southeast—(Andhra Pradesh)				
		East—(West Bengal)				
		Southwest—(Maharashtra)				
		Central—(Madhya Pradesh)				
		West—(Rajasthan)				
9	*Naja naja*	Hindustan Park (Kolkotta, West Bengal)	Haffkines	LD50, Poteolytic and hemolytic inhibitory activities	This study suggests developing region-specific antivenoms for the effective management of snakebites.	[52]
		Irula Snake Catchers (Chennai, Tamilnadu)				
		Haffkine Institute (Mumbai, Maharashtra)				
10	*Naja naja*, *Naja oxiana*, and *Naja kaouthia*	Himachal Pradesh, West Bengal, Mizoram, Assam, Maharashtra, Tamil Nadu (Irula) and Arunachal Pradesh	VINS, PSAV	WB, IAC	Antivenomics indicated that low-molecular-mass proteins such as PLA2 and 3FTXs were recognized poorly by the antivenom.	[53]
11	*Echis carinatus*	Maharashtra (West India)	BSAV, PSAV, VINS, and Haffkine	ELISA, WB, LD_50_, ED_50_	The detection and binding efficacies of antivenoms seems to vary among all of the snake venoms tested.	[48]
12	*Echis carinatus*	Tamil Nadu, Goa and Rajasthan	VINS	size-exclusion chromatography	Compared venom collected from Goa and Rajasthan; the *E. carniatus* venom collected from Tamil Nadu resulted in the formation of more venom–antivenom complexes, indicating binding efficacy.	[54]
13	*Echis carinatus*	Goa and Tamil Nadu	BSAV	IAC	Low-molecular-mass proteins, especially disintegrins, present in the venom showed poor binding to the antivenom tested.	[55]
14	*Echis carinatus*	Southern India—Tamil Nadu, Irula snake catcher’s society	BSAV, PSAV, Virchow	ELISA, WB, IAC, and pro-coagulant activity	The antivenoms poorly recognized the low-molecular-mass proteins (<20 kDa) present in *E. carinatus* venom.	[42]
15	*Echis carinatus sochureki*	Rajasthan (Northwest India)	BSAV, PSAV, VINS, and Haffkine	ELISA, WB, LD_50_, ED_50_	The detection and binding efficacies of the antivenoms seems vary among all of the snake venoms tested.	[48]
16	*Daboia russelii*	Eastern India (Nadia and Burdwan District, West Bengal)—Calcutta Snake park	BSAV, PSAV, Virchow, and BE	ELISA, WB, IAC	All of the antivenoms failed to recognize low-molecular-mass proteins (<20 kDa).	[56]
17	*Daboia russelii*	Southern India-Tamil Nadu-Irula snake catcher’s society	BSAV, PSAV, Virchow, and BE	ELISA, WB, IAC, and neutralization of enzyme activities and pharmacological properties	Poor recognition of the low-molecular-mass protein(<20 kDa) of *Naja naja* venom from Western parts of India by all the antivenoms.	[57]
18	*Daboia russelii*	Southern India-Tamil Nadu-Irula snake catcher’s society	Haffkine, VINS, BE, and PSAV	ELISA, WB, LD_50_, ED_50_, and IAC	The immunological cross-reactivity was different towards all of the antivenoms.	[33]
19	*Daboia russelii*	Western India-Haffkine Institute, Mumbai	VINS and PSAV	ELISA, WB	Both the antivenoms exhibited poor cross-reactivity towards low-molecular-mass proteins (<18 kDa) in the crude venom. The study also demonstrated that monovalent antivenoms are better than polyvalent antivenoms.	[41]
20	*Daboia russelii*	North- Punjab	BSAV, PSAV, Haffkine, and VINS	ELISA, WB, LD_50_, ED_50_	The antivenoms showed poor immunological cross-reactivity against all of the venoms used, indicating the need for pan-India effective antivenoms.	[58]
		South-(Tamil Nadu)				
		Southeast-(Andhra Pradesh)				
		East-(West Bengal)				
		Southwest-(Maharashtra)				
		Central-(Madhya Pradesh)				
21	*Daboia russelii*	Tamil Nadu region (South India)	VINS	ELISA, LD_50_, procoagulant activity and neutralization	Compared to high-molecular-mass venom proteins, the low-molecular-mass proteins were poorly recognized by the antivenom.	[59]
22	*Bungarus caeruleus*	Southern India-Tamil Nadu-Irula snake catcher’s society	BSAV, PSAV, and BE	ELISA, WB, IAC	Poor recognition of low-molecular-mass proteins (<15 kDa) such as three-finger toxins and phospholipase A_2_ by the antivenoms.	[40]
23	*Bungarus caeruleus*	South-eastern India, unspecified locales of India, supplied by Latoxan (France)	VINS, Neuro Polyvalent Antivenom (NBAV), and Bungarus candidus Monovalent Antivenom	ELISA, LD_50_, ED_50_	All venoms showed better immuno-reactivity profiles towards VINS antivenom. Also, compared to venom from Pakistan and Sri Lanka, Indian venom was effectively neutralized by the antivenoms.	[60]
24	*Bungarus sindanus*	Bikaner, Rajasthan	Haffkine and PSAV	ELISA, WB, LD_50_, and ED_50_	The antivenom effectively neutralized *B. caeruleus* venom, whereas *B. sindanus* and *B. romulusi* showed poor cross-reactivity profiles towards the antivenom.	[61]
	*Bungarus sindanus*	Pune, Maharashtra				
	*Bungarus caeruleus*	Pune, Maharashtra				
	*Bungarus romulusi*	Bannerghatta, Karnataka				
25	*Bungarus caeruleus*	Punjab (North India)	BSAV, Haffkine, PSAV, and VINS	ELISA, WB, LD_50_, ED_50_	All the antivenoms recognized the venom antigenic epitopes in the ‘big four’ snake species and showed varied immunological cross-reactivity towards venom from other species.	[48]
26	*Bungarus sindanus*	Rajasthan (Northwest India)				
27	*Bungarus fasciatus*	West Bengal (East India)				
28	*Naja kaouthia*	Arunachal Pradesh (Northeast India)				
29	*Naja kaouthia*	West Bengal (East India)				
30	*Naja kaouthia*	North East India (Assam—Guwahati and Jamurighat)	VINS	WB, IAC	The VINS polyvalent antivenom could not recognize the few three-finger toxins present in *Naja kaouthia* venom.	[62]
31	*Naja kaouthia*	East India (Kolkata, West Bengal, and Arunachal Pradesh)	BSAV, Haffkine, PSAV, VINS, and Thai monovalent *N. kaouthia* antivenom (QSMI)	LD_50_, ED_50_, ELISA	The study concluded that intraspecies venom variation affects antivenom efficacy.	[63]
32	*Naja kaouthia*	Eastern India-(Burdwan District, West Bengal)-Calcutta snake park, Kolkata	BSAV, PSAV	ELISA, WB, IAC	Both antivenoms showed poor immunological cross-reactivity profiles towards the low-molecular mass proteins (<20 kDa) present in *N.kaouthia* venom	[35]
32	*Naja kaouthia*	Assam	BSAV, PSAV, Virchow, VINS	ELISA, WB	The polyvalent antivenoms poorly recognized the low-molecular-mass proteins (<15 kDa) present in *N.kaouthia* venom from northeastern India.	[64]
34	*Naja kaouthia*	North-East India and Bangladesh	VINS, Haffkine, and BSAV	WB, ED_50_, LD_50_, IAC	Antivenoms showed better immunological cross-reactivity towards high-molecular-mass components. VINS antivenom poorly recognized low-molecular-mass proteins.	[32]
35	*Trimeresurus malabaricus*	Western Ghats of India-Kerala	VINS, PSAV, Virchow	ELISA, WB	Compared to Russell’s viper venom, all of the antivenoms showed poor immunological cross-reactivity towards Malabar pit viper venom proteins.	[65]

Median Lethal Dose (LD_50_); Median Effective Dose (ED_50_); Western blotting (WB); Immunoaffinity chromatography (IAC); BSAV—Bharat Serums and Vaccines Ltd.; PSAV—Premium Serum and Vaccines Pvt., Ltd.; VINS—Vins Bioproduct Limited Virchow-Virchow Biotech Pvt. Ltd.; Haffkine—Haffkine Biopharmaceuticals Corporation Ltd.; BE—Biological E Limited.

## 5. General Strategies for Determining the Omes and Omics of Snake Venom

The venom proteome composition of medically important snake species and the antivenom efficacy can be determined through several strategies, a few of which are discussed below: 

### 5.1. Snake Venom Proteomics

During the late twentieth century, the identity of venom proteins was established through conventional biochemical assays and analytical strategies [66]. One of the major drawbacks of these strategies was that they failed to determine the presence of non-enzymatic proteins in snake venom. The advent of electrospray ionization [67] and matrix-assisted laser desorption ionization mass spectrometry [68,69] ionization techniques have helped to delineate the venom proteome in a different way. Based on these ionization strategies, several mass spectrometers have been extensively used to characterize the venom proteome of several snake species, including the ‘big four’ snake species in India [12,48,65,70,71,72,73,74,75,76]. The venom proteome of several snake species, including the ‘big four’ snake species in India, has been explored using various mass spectrometry-based proteomics workflows [33,35,40,42,48,77]. Venom proteome profiling can be achieved through bottom-up and top-down strategies. The latter involves intact mass measurement, the estimation of disulfide linkages, and the identification of post-translational modifications. At the same time, bottom-up proteomics involves separating venom protein components using orthogonal separation strategies, including reversed-phase chromatography and SDS-PAGE analysis. The resolved proteins are then subjected to enzymatic digestion using trypsin, chymotrypsin, and V8 protease, and the raw data can be collected using various mass spectrometers [50]. Finally, the identity of the proteins can be established through data analysis using appropriate databases. Nevertheless, several limitations exist, including the non-availability of protein sequence information in public repositories. However, this information is crucial in determining the effectiveness of commercially available antivenoms. 

### 5.2. Genomics and Venom Gland Transcriptomics

By using prediction algorithms and homology searches (using reference sequences), translated genes (exome) can be determined through whole genome sequencing [78]. This approach can also be helpful in estimating the molecular basis of adaptation and the evolution of various snake species [79,80]. The differences in venom composition and genes’ identity in coding venom–protein sequences can easily be established through genomics rather than transcriptomics alone [81,82]. Genome sequencing can also determine structural variations such as inversions, insertions, deletions, duplications, and rearrangements [83]. Studying the snake genome not only helps delineate the identity of venom-coding genes but also helps in designing antivenoms towards the antigenic epitopes identified. For example, the genome of several snake species, including the Indian cobra, has been published [79,80,82,84,85], and this information can be utilized for designing synthetic antivenoms. Genomic information might be useful in generating humanized recombinant antivenoms [82]. This might also be useful in generating species-specific antivenoms to counteract the life-threatening envenomation effects of these snake species. 

### 5.3. Immunological Cross-Reactivity Studies and Antivenomics

**Figure 1:**
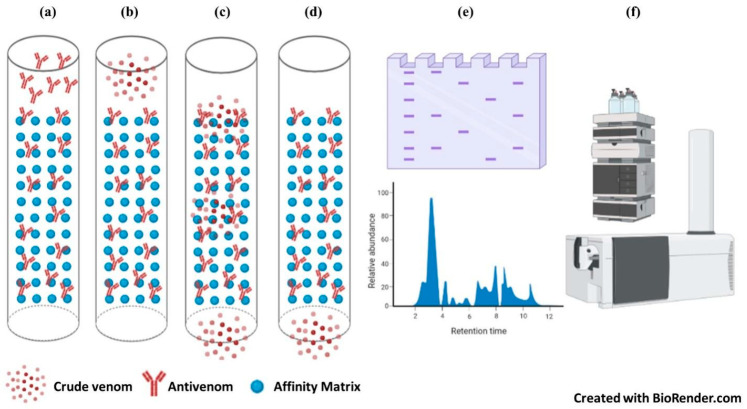
Antivenomics workflow. (**a**) Incubation of the antivenom with the matrix (sepharose or agarose beads). (**b**) Addition of crude venom. (**c**) Elution of unbound venom proteins. (**d**) Elution of bound venom proteins. (**e**) Resolve the bound and unbound fractions through orthogonal separation strategies (upper panel—SDS-PAGE analysis; bottom panel—reversed-phase HPLC analysis). (**f**) The collected fractions are then subjected to mass spectrometry-based proteomics for the identification of antivenom-bound and unbound proteins.

The complex toxin arsenal of various snake species could be determined through venomics studies. The data obtained through snake venomics might be useful in improving antivenom production strategies through immunological cross-reactivity studies. This will also be helpful in determining the cross-reactivity and binding potential of antivenoms toward the homologous proteins present in various snake species [86]. Since antivenoms are the main form of treatment for snakebite envenomation, efficient and reliable strategies must be constructed to assess the therapeutic potential of antivenoms [87]. Several in vitro preclinical studies include end-point titration and avidity ELISA, immunoblotting, and immunoaffinity chromatography approaches (Table 3). Immunoblotting and end-point titration ELISA are being used widely to assess the cross-reactivity potential of antivenoms. Immunoblotting is a qualitative method, whereas, through ELISA, we can effectively quantify the amount of antivenom required to detect and bind to venom antigenic epitopes. However, the venom antigens that are specifically neutralized by the antivenom need to be explored; this is a major limitation of the previously mentioned approaches. To overcome this problem, several antivenomics strategies have been outlined to effectively determine antivenom-bound and unbound venom protein constituents. This immunoaffinity-based ‘antivenomics’ approach can give more accurate quantitative information on the binding and neutralization potential of antivenoms for the treatment of snakebite envenomation [86,88] (Figure 1). 

## 6. Alternatives to Antivenom

### 6.1. Aptamers

Since there are a lot of concerns associated with antivenom including the storage, production, purification, and efficacy of antivenoms, finding alternative strategies is an urgent need. Due to this, many alternatives to antivenoms, including developing and designing toxin-specific oligonucleotide-based aptamers, are currently being considered [89]. Aptamers are single-stranded DNA or RNA oligonucleotides that have high specificity and affinity towards the target [90]. They are used for a wide variety of applications [91], and one such polynucleotide aptamer, Pegaptanib, which inhibits vascular endothelial growth factor, is typically used for treating age-related macular degeneration [92]. Using Systematic Evolution of Ligands by Exponential enrichment (SELEX) technology, aptamers are selected from a library of synthesized oligonucleotides [93]. Aptamers are preferred to antibodies because of their low immunogenicity, low cost, superior shelf-life, thermal stability, smaller size, biocompatibility, and easier production strategies [94]. Reports have shown that aptamers neutralize snake venom, cone snail, and scorpion toxins [90,95,96,97,98]. Most of the studies using aptamers are limited to purified snake venom proteins. However, further studies are required to prove the efficacy of these aptamers in neutralizing entire venom protein constituents. Table 4 shows a summary of aptamers designed for the treatment of various snake venom toxins. Our detailed review on the application of aptamers as anti-dotes or as diagnostic tools indicates that the studies on the use of aptamers in the literature at the time of writing were limited to only a few species. For example, most of the aptamers were designed to target low-molecular-mass toxins, especially the neurotoxins from various krait species [90,96,99,100]. ssDNA aptamers designed to fight against snake venom serine proteases such as ancrod and batroxobin from *C. rhodostoma* and *B. atrox* inhibited venom-induced coagulopathy through reducing the consumption of fibrinogen and plasma clotting activities [93]. Thus, the use of these technologies can be expanded in order to develop aptamers for other medically important snake species. 

### 6.2. Camel Antivenoms

Another alternative strategy to conventional antivenom production is to generate single-domain antibodies from the camel H-chain (V_H_H) antibody [102,103]. Contemporary antivenoms are generally based on IgG-type antibodies and Fab and F(ab)2 fragments. All of these types of antibodies have a few drawbacks, including their limitations in penetrating tissues or blood to reach their target [104]. Moreover, the presence of Fc regions in whole IgG-type antivenoms might induce severe adverse effects, including anaphylaxis and serum sickness [105,106,107,108]. Since camelid immunoglobins are devoid of light chains, they tend to interact with the antigen only through available single variable (V) regions. Hence, these antibodies isolated from camels are known as V_H_H or nanobodies. Therefore, these types of antibodies can be easily screened and isolated even through phage display techniques [102,103]. Moreover, the camelid IgGs are known to be less immunogenic and highly thermostable, and they feebly activate the complement cascades [109]. Several studies have shown that camelid antibodies effectively neutralize scorpion [110,111,112], Australian paralysis tick [113], and snake venom [103,104,114,115,116]. In one study, the use of camelid antivenom was found to be effective in neutralizing the hemorrhage, coagulant, lethal, and local effects of *Echis carinatus sochureki* venom [115]. Similarly, hemorrhagic effects induced by the venom of *Echis ocellatus* [104] and α-cobratoxin (from *Naja kaouthia* venom)-induced lethal effects [103] were found to be neutralized effectively using camelid antivenoms. All of these reports indicate that, compared to equine antivenoms, camelid antivenoms may be an effective alternative strategy for combating snake venom-induced complications. 

### 6.3. Phage Display

Phage display [117,118] is a powerful molecular biology technique used for the screening of peptides or antibodies against target antigen. Inserting the DNA of the desired protein or peptide into the gene of the bacteriophage coat protein allows one to display these molecules on the surface of the filamentous bacteriophage. Thus, a large library of phages displaying proteins or peptides of interest can be developed and used to interact with a target protein [117]. Several groups have investigated the feasibility of using phage display to develop recombinant antibodies or peptides (Table 5) against snake venom [119], which could lead to the discovery of therapeutics which are advantageous over conventional animal-derived antivenoms. Recently, an 8-mer peptide that exhibited binding to α-cobratoxin and subsequently the inhibition of nicotinic acetylcholine receptors in Xenopus oocytes by α-cobratoxin was identified using phage display and deep sequencing [120]. These peptide-based antivenoms could act as an alternative to animal-derived antivenoms, but their efficacy and in vivo pharmacokinetics should be thoroughly evaluated. A cocktail of peptides against the major venom toxins could be effective in binding and neutralizing venom components. Here, we have reviewed the most recent advancements in developing alternative strategies through phage display to generate improved antivenoms. 

Contemporary treatments for envenomation use animal-derived polyclonal whole IgG molecules, which have several disadvantages. Hence, approaches using peptides and other small molecule-based antivenoms are considered as alternative strategies. As opposed to polyclonal [126] antibodies, monoclonal antibodies or antibody fragments have been shown to serve as better options in envenomation treatments. Comparisons between polyclonal chicken IgY and horse-derived antibodies and scFv (single-chain fragment variable) are also being made. A study showed that polyclonal IgY generated to fight against *Naja naja atra* venom in chicken was able to recognize the low-molecular-mass proteins present in *Naja naja atra* venom, but horse-derived antibodies recognized only proteins with a molecular mass greater than 22 kDa. They also generated antibody libraries for monoclonal scFv (single-chain variable fragment) antibodies against *Naja naja atra* venom, which recognized low-molecular-weight *Naja naja atra* proteins. In addition, they checked their effect in vivo and found that polyclonal IgY provided full protection, whereas the neutralization of *Naja naja atra* venom using monoclonal scFv antibodies was only partial in mice [121]. Additional tests were performed using polyclonal IgY antibodies to fight against *Trimeresurus stejnegeri* venom produced by immunizing chicken with *T. stejnegeri* venom, and scFv antibodies were generated using phage display. The authors also compared the binding affinities of both IgY and scFv antibodies and found that scFv antibodies could recognize *T. stejnegeri* venom proteins, also showing some cross-reactivity towards *Trimeresurus mucrosquamatus* proteins. IgY antibodies provided complete protection against *T. stejnegeri* venom in mice, whereas a combination of scFv antibodies reduced venom-induced mortality among mice [122].

Polyclonal antibodies have limited neutralization capacities due to the low immunogenicity of some snake venom toxins. A combination of polyclonal and human monoclonal antibodies has shown to be effective in circumventing this problem. A recent report has shown that phage display can be used to generate recombinant monoclonal antibodies to fight against snake venom from two different snakes. A study was performed to generate monoclonal antibodies using phage display by cross-panning against two different cobratoxin. The selected monoclonal antibodies showed broadly neutralizing potential against three different cobras: *Naja nigricollis*, *Naja mossambica*, and *Naja melanoleuca*. Hence, this study demonstrates the possibility of developing broadly neutralizing monoclonal antibodies by using a single antigen, but these antibodies could be capable of neutralizing homologous toxins of the original antigen used for phage display [123]. Recently, the light chain shuffling of antibodies, where the heavy chain of the antibody is retained but alternative light chains are explored, has been proposed as another method to generate better antivenoms. Recently, a study focused on developing a cross-reactive antibody by cross-panning the light chain-shuffled scFv antibody with two different toxins—α-cobratoxin and α-elapitoxin—from the venom of *Dendroaspis polylepis*. The selected scFv antibodies exhibited improved affinity and increased cross-neutralization in vitro and in vivo compared to the parent antibody against the α-neurotoxins of the elapid venoms of snakes belonging to the following genera: *Dendroaspis*, *Ophiophagus*, *Bungarus*, and *Naja* [124].

**Figure 2:**
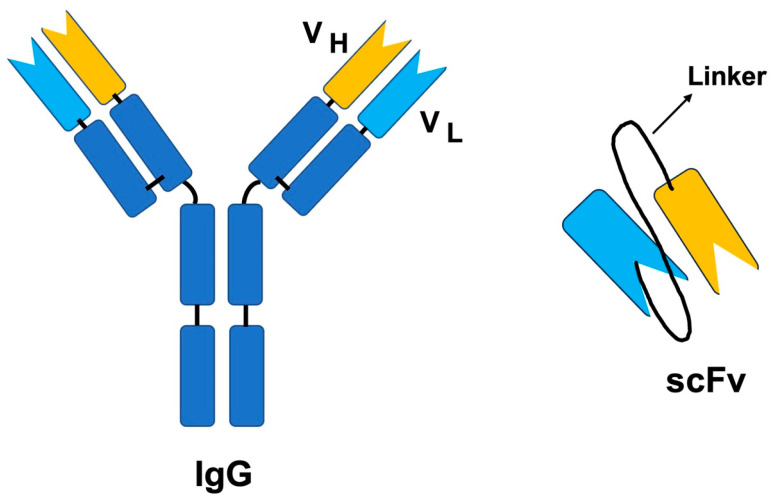
Whole Immunoglobuling IgG and single-chain (scFv) antibody fragment. Adapted from https://blog.addgene.org/antibodies-101-single-chain-fragment-variables-scfvs (accessed on 20 May 2023).

**Figure 3:**
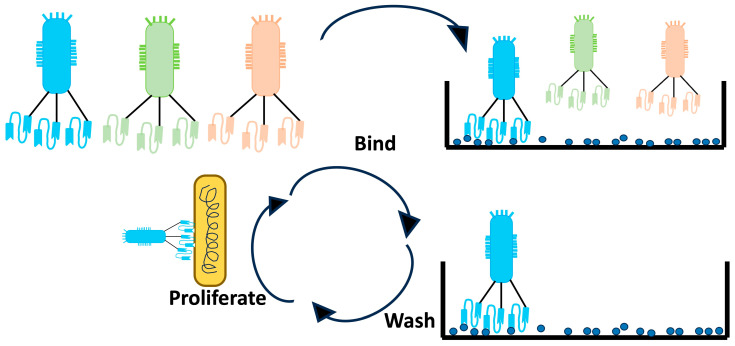
Phage display with scFv on the phages are used to bind to the target antigen. High-binding phages are further amplifed to enrich these bound phages. Adapted from https://blog.addgene.org/antibodies-101-single-chain-fragment-variables-scfvs (accessed on 20 May 2023).

Unlike the full-length antibody molecule, ScFv is a smaller version of the antibody, where the variable light chain and variable heavy chain of the Fab fragment are retained. The V_H_ and V_L_ are connected by a peptide linker (Figure 2). There are several advantages of selecting scFv over IgGs, including the benefits related to size. The small size of scFv allows for the generation of these molecules in bacterial expression systems, while antibodies require mammalian systems. Additionally, they are small enough to be displayed on the phage; thus, they are easier to screen using the phage display method (Figure 3). Moreover, since phage display is an in vitro technique, animal immunization can be avoided. Faster clearance from the blood, better diffusion into tissues, and less immunogenicity due to the lack of Fc region are some of the most important advantages of using scFv. The Fc region can activate Fc receptor-expressing cells, thus resulting in a massive release of cytokines and leading to toxicity [127].

A recent study has shown that a monoclonal scFv antibody generated to fight against the cytotoxins of *Naja atra* venom was able to neutralize and prevent cytotoxicity in C2C12 myoblast cells [126]. Similarly, Cro-Bothrumabs is a human scFv fragment that recognizes venoms of both bothrops and crotalus. This is the first report involving a polyvalent antivenom fighting against *Bothrops jararacassu* and *Crotalus durissus terificus*. The antibody fragment was shown to be capable of neutralizing both of the venoms in both in vitro and in vivo experiments [125].

Thus, phage display is a promising technique that can be used to develop better antivenoms due to its specificity and lack of adverse effects compared to the techniques used to develop animal-derived antivenoms. However, to date, there is no published data pertaining to India available on the use of phage display for developing better antivenoms.

### 6.4. Small-Molecule Inhibitors

**Figure 4:**
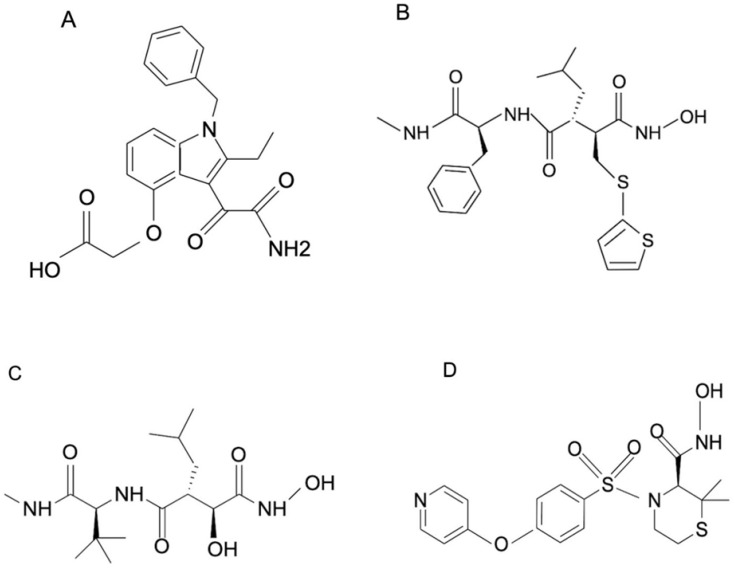
The chemical structures of the small-molecule inhibitors: (**A**) varespladib, (**B**) batimastat, (**C**) marimastat, and (**D**) prinomastat. All chemical structures were drawn using ChemDraw^®^ J S.

Due to the above-mentioned limitations of antivenoms, including the inability to neutralize low-molecular-weight toxins of venom, which can have deleterious effects, the repurposing of small-molecule inhibitors against these toxins has become a major area of research for the development of better treatment strategies for snake envenomation. There are various studies showing the inhibitory action of these small-molecule inhibitors on various toxins like phospholipase A_2_ [128] and metalloproteases in venom (Table 6). The most studied among them is varespladib (LY315920), which is a non-specific inhibitor of mammalian sPLA_2_ that has previously been used to treat acute coronary syndrome [129] but failed in a Phase II clinical trial. In 2016, Lewin et al. demonstrated that varespladib could inhibit PLA_2_ from the snake venoms of several snake species from six different continents [130]. Three other compounds—prinomastat, batimastat, and marimastat (Figure 4)—had a similar effect on the action of venom metalloproteases. These molecules were initially used to treat tumors but were shown to inhibit the metalloproteases of snake venom [131]. 

A recent study has shown that varespladib was able to inhibit the anticoagulant and procoagulant activities of venom. A study conducted by Xie et al. found that varespladib might also interfere with other toxins since, in their study, they observed that varespladib could partially abrogate the procoagulant activities of venom. Procoagulant activities are caused by the action of proteases [132]. Further studies are required to assess whether varespladib could affect other venom toxins also [133]. This could be an important finding since both PLA_2_ and snake venom metalloproteases are responsible for necrosis leading to tissue damage after envenomation, and these effects may not be neutralized effectively due to delays in the administration of antivenoms. In these scenarios, alternate inhibitors could be effective as a first-aid treatment prior to antivenom administration, as shown in previous studies [134,135]. It is still unknown whether varespladib can abrogate the effects of procoagulant toxins present in other snake venoms since the aforementioned study was performed only with the venom of a few snake species.

**Table 6:** Studies on small-molecule inhibitors against snake venom.

SI No.	Small-Molecule Inhibitor	Snake Species	Protein	Study Design	Ref.
1	Varespladib	*Bothrops asper*, *Calloselasma rhodostoma*, *Deinagkistrodon acutus*, *Daboia russelii*, *Echis carinatus*, *Echis ocellatus*, and *Oxyuranus scutellatus.*	Phospholipase A_2_	In vitro	[133]
2	Varespladib	*Daboia siamensis*	Phospholipase A_2_	In vitro	[136]
3	Batimastat	*Crotalus atrox*	Group I (PI) metalloprotease	In vitro and in silico	[137]
4	Marimastat	*Crotalus atrox*	Group I (PI) metalloprotease	In vitro and in silico	[137]
5	Varespladib	*Naja ashei*, *Naja katiensis*, and *Naja nubiae*	Phospholipase A_2_	In vitro	[138]
6	Prinomastat	*Naja ashei*, *Naja katiensis*, and *Naja nubiae*	Phospholipase A_2_	In vitro	[138]

Similarly, another study assessed the effectiveness of antivenom alone and in conjunction with varespladib on the neurotoxicity induced by Chinese *D. siamensis* venom. The study was performed in vitro by isolating the chick biventer cervicis nerve muscle preparation in an organ bath to assess the effect of antivenom alone and in combination with varespladib on the neuromuscular blockade induced by the venom. The researchers found that neurotoxicity cannot be reversed by the monovalent antivenom. But the pre-incubation of venom with varespladib abrogated the neurotoxic and myotoxic activities of the venom. 

However, when both venom and varespladib were given in combination, it did not result in an increased inhibitory effect on neurotoxicity or myotoxicity [136]. Additionally, other studies showing the inhibitory effect of varespladib on neurotoxic PLA_2_s have also been performed. In 2020, Gutierezz et al. showed that varespladib and methyl varespladib were able to rescue mice injected with venoms of *Notechis scutatus*, *Crotalus durissus terrificus*, *Bungarus multicinctus*, and *Oxyuranus scutellatus*, where pre-synaptically acting neurotoxic PLA_2_s with different quarternary structures were major toxins in these venoms [139]. Hence, this study also aimed to check whether varespladib was able to inhibit the PLA_2_s with a different quaternary structure. A recent study by Tan et al. in 2022 demonstrated the effect of varespladib on the neurotoxic activities of the five major kraits of Asia: *B. caeruleus*, *B. sindanus*, *B. candidus*, *B. fasciatus*, and *B. multicinctus*. These reports indicate the efficacy of varespladib in combating the toxic effect of venom PLA_2_ and thus could be used as an adjunct treatment for snake envenomation [140]. Thus, several in vitro and in vivo studies have been conducted to evaluate the preclinical efficacy of varespladib. Following this, a study protocol, BRAVO (Broad-spectrum Rapid Antidote: Varespladib Oral for snakebite), was recently designed to evaluate the clinical effects of varespladib-methyl. This is the first international clinical trial of a treatment for snakebite envenomation [141]. This study was designed to compare and evaluate the efficacy of varespladib-methyl plus standard of care with placebo plus standard of care in patients affected by snakebite envenomation.

Like varespladib, there are matrix metalloprotease inhibitors that could also prove to be promising against snake envenomation since they can inhibit snake venom metalloproteases. These molecules were initially developed for treating cancer, but they failed in clinical trials [142,143,144]. Recently, they were shown to inhibit the SVMP activity of western diamondback rattlesnake venom [137]. From all of these studies, it is likely that small-molecule inhibitors could be used as a form of complementary or adjuvant therapy in conjunction with antivenom administration. Furthermore, these molecules could be used as a first-aid treatment in a field setting where antivenom administration could be time-consuming due to a lack of availability regarding healthcare facilities. This could prevent delays in treating snakebite victims and thus avoid significant morbidity and mortality rates due to envenomation. Small-molecule inhibitors are usually targeted against a particular venom toxin, but since the venom components act in a synergistic way, a combination of these inhibitors might be appropriate to use as an adjuvant or first-aid treatment before antivenom administration.

From an Indian perspective, studying the effect of small-molecule inhibitors could prove promising. About half of the snakebite deaths occurring in India are due to envenomation by Russell’s viper, the venom of which has many toxins that affect the coagulation mechanism of the victim. PLA_2_ is the most abundant toxin of RV venom [57], and there are many isoforms of this toxin [31] in the venom of a single snake species. With a low molecular weight, small size, and, consequently, being less immunogenic, these toxins are not always effectively neutralized by the polyvalent antivenoms developed in India. Hence, alternate strategies like exploring the effect of the small-molecule inhibitors of these toxins should be developed to fight against limitations of antivenom.

### 6.5. Natural Products 

Time is an essential factor governing the pathophysiology of envenomation, and most of the snakebite cases are reported from rural areas, where antivenom facilities are scarce and hospitals are usually out of reach [23]. In these scenario, using a traditional plant-based treatment as an alternative or supporting therapy to circumvent an antivenom’s limitations is of great importance. 

Traditional medicines make use of plant extracts or derived compounds like secondary metabolites to treat snakebites. These products can be procured with ease, are relatively economical, and bring fewer administrational complications [24]. The therapeutic applications possessed by plants can be of two types: On one hand, the active components present in crude plant extracts can be used as inhibitors for snake venom toxins. On the other hand, the chemical structure of active constituents can be modified to target certain toxins in venom [145]. The pathways targeted by the venom toxins can be used as a target to develop inhibitors from plant products, and such an effort was shown in a study in which aqueous stem bark extracts of *Mangifera indica* were used to inhibit Group IA sPLA_2_ (phospholipase A_2_), which are enzymes that regulate the release of inflammatory mediators via the arachidonic acid pathway, thereby playing an important role in inflammation at the bite site [146]. The active constituents present in the plant extracts exert their action by interfering with the binding sites of the substrate or enzyme or by chelating the metal ions needed for enzymatic activity, inhibiting the enzyme [7]. The separation of the active components behind neutralization is one of the major limitations in developing the natural product-based inhibitors of snake venom. Several studies have been successful in isolating bioactive compounds from plant extracts. However, the validation of their toxicity profiles is a major hindrance to its development as a drug candidate [147]. 

Medicinally important plants can be screened to find inhibitors that act against different components present in snake venom and isolate the lead molecule responsible for the inhibitory effects [148]. Previous studies have demonstrated that various parts of plants can be used to treat snakebites and their associated symptoms. Leaves are commonly used to treat snakebites. Among the botanical family of plants, the most studied family regarding potential snake venom-inhibiting substances is the Fabaceae, followed by the Zingiberaceae, Salicaceae, and Asteraceae families [24]. Regarding secondary metabolites derived from plants with snake venom-inhibiting properties, studies have shown that phenolic compounds are the major class of compounds that contribute to snake venom neutralization [24]. Natural products can be designed to inhibit various toxin families present in snake venom (Table 7), as these are the class of proteins that act upon different physiological phenomena in the victim, leading to clinical manifestations such as local tissue damage [149,150]. 

A lot of plant species have reportedly shown anti-PLA_2_ activity. One of the main drawbacks of contemporary antivenoms is that the low-molecular-mass toxins in the venom are poorly recognized compared to high-molecular-mass components (Table 3). As these proteins are less immunogenic, antibodies designed to fight against these proteins are not efficiently generated, leading to low neutralization. One such low-molecular-mass protein is PLA_2_, which is responsible for inflammation and pain in bitten areas [151]. Plants used to treat inflammatory disorders could be screened for inhibitors of the enzyme phospholipase A_2_. One such inhibitor is Aristolochic acid, which is extracted from the plant root of the Aristolochia species and has been reported to inhibit PLA_2_ and L-amino acid oxidase (LAAO) components in snake venom [152,153]; however, due to the DNA-binding property of this molecule, it is considered to be carcinogenic. Hence, a modified semi-synthetic derivative of the molecule is used for inhibition [152]. One study reported the neutralization of the PLA_2_ enzyme from Cobra and Russell’s viper venom using a compound isolated from neem leaf extract, AIPLAI (*Azadirachta indica* PLA_2_ inhibitor) [154]. A later study demonstrated the use of neem plants for treating poisonous bites, including snakebites, by the Irula tribes of Walayar valley, South India, who practice herbal medicines using the indigenous plants found in the region to treat different ailments [155]. This information substantiates the previous research findings on AIPLAI. Further studies on the aqueous root extract of *Mimosa pudica* showed inhibitory activity towards the hyaluronidase and protease enzymes of *Naja naja*, *Echis carinatus*, and *Daboia russelli* venom [156]. The aqueous extract of *Morus alba* leaves has been shown to inhibit the systemic effects and local tissue damage induced by Russell’s viper venom. The extract acts against venom metalloproteinases (SVMP), wherein the metal ion chelation by the extract leads to the inactivation of the enzyme [157]. However, the active components responsible for these activities were not isolated. Many studies have been performed with compounds isolated from plants like secondary metabolites; one particular study involving a compound, quercetin-3-O-α-rhamnoside, from the Indian species *Euphorbia hirta* (Euphorbiaceae) significantly inhibited the PLA_2_ enzyme from *N. naja* by 93%. It also inhibited hyaluronidase and hemolytic activity [158]. Regarding plants endemic to India, a *H. indicus* (Apocynaceae)-derived compound, 2-hydroxy-4-methoxy benzoic acid, has shown inhibitory effects on *N. kaouthia*, *O. hannah*, *D. russelii*, and *E. carinatus* venom. The compound effectively inhibited *D. russelii* venom-induced inflammation and hemorrhage and coagulant effects in mouse models [159,160,161]. Apart from Flavonoids, Alkaloids, and secondary metabolites, a glycoprotein isolated from a *Withania somnifera* plant, WSG, has shown inhibitory potential towards cobra venom toxic PLA_2_ [162]. This is an example of mimicking the structure analogy of natural PLA_2_ inhibitors present in snake plasma. The glycoprotein WSG is similar to the α-chain of the phospholipase inhibitors(PLIs) found in snake plasma [163,164]. 

Considering the vast opportunities plant products offer, in silico studies using molecular docking could be employed to identify the bioactive components that help bind to the active site of the toxins in snake venom and study their interaction mechanisms. This could help in designing modified plant compounds with better binding ability and hence improved neutralization potential [165,166].

## 7. Conclusions

Since geographical venom variation contributes to antivenom efficacy, from a regional perspective, there is a pressing need to develop antivenom. Antivenom availability is limited in certain countries, including Sri Lanka, Bangladesh, Pakistan, and Nepal. Hence, these countries depend on Indian polyvalent antivenoms generated using the venom collected from the big four snakes of India. When numerous publications report the ineffectiveness of Indian antivenoms even towards Indian snakes, it is alarming not only for Indian citizens but also for those in neighboring countries that depend on Indian antivenoms to treat snakebites. Preclinical studies have indicated the inefficiency of Indian polyvalent antivenoms in detecting and binding to the venom epitopes present in the snake species of other countries, and few studies agree with the cross-neutralization of homologous venom proteins. However, their efficacy towards proteins of a heterologous nature is still a concern, and more studies need to be performed to validate the results of prior studies. Additionally, several reports have indicated that, in addition to venom from the ‘big four’ snakes, venom from other medically important snake species needs to be included in the horse immunization mixture when developing antivenoms. Since the morbidity and mortality rates are high, effective measures need to be outlined to reduce the burden imposed by this neglected disease. To achieve this, several alternatives have been proposed, including synthetic antibodies, repurposed drugs, and small-molecule inhibitors; however, all of these potential treatments need to be approved in clinical trials. Some of the major drawbacks of antivenoms include the fact they do not completely neutralize the local effects of envenomation, they have low neutralization capabilities with respect to low-molecular-weight proteins, and the fact that serum sickness towards the host antigen present in the antivenom can occur. These strategies can also be implemented along with antivenom treatment so that it will slow down some of the effects induced after SBE. Nevertheless, clinical trials should be performed to test the efficacy of administering these treatments in conjunction with antivenom. To conclude, since comprehensive expertise is not available under a single roof, thorough inter-institutional and intra-institutional collaboration within the country and outside India is needed to explore the potential of implementing these alternatives to counteract snakebite envenomation. 

## Figures and Tables

**Table 1:** Medically important snake species of India and antivenom availability (Retrieved from the Snakebite Data Information portal—World Health Organization) [5]. Categories 1 and 2 indicate highly venomous snakes with the highest medical importance and secondary medical importance, respectively.

SI NO.	Taxonomic Family	Species Name	Common Name	Geographical Distribution in India	Antivenom Available and Licensed in India	Nature of Available Antivenom (s)	Antivenom Manufacturing Countries
	Category-1						
1	Elapidae	*Bungarus caeruleus*	Indian krait	Throughout	Yes	Polyvalent antivenom	India, Pakistan
2		*Naja naja*	Indian cobra	Throughout	Yes	Polyvalent antivenom	India, Pakistan
3		*Naja kaouthia*	Monocellate cobra	Northeast	No	Monovalent and Polyvalent	Myanmar, Thailand, Vietnam
4	Viperidae	*Daboia russelii*	Russell’s viper	Throughout	Yes	Polyvalent antivenom	India, Pakistan
5		*Echis carinatus*	Saw-scaled viper	Throughout	Yes	Polyvalent antivenom	India, Pakistan, Iran, Uzbekistan, Spain
6		*Hypnale hypnale*	Hump-nosed pit viper	Southwest	No	NA	-
	Category-2						
7	Elapidae	*Bungarus bungaroides*	Northeastern hill krait	Northeast	No	NA	-
8		*Bungarus fasciatus*	Banded krait	Northeast	No	Polyvalent and monovalent antivenoms	Thailand, Indonesia
9		*Bungarus lividus*	Lesser black krait	Northeast	No	NA	-
10		*Bungarus niger*	Greater black krait	Northeast	No	NA	-
11		*Bungarus sindanus*	Sind krait	Northwest	No	Polyvalent antivenom	Pakistan
12		*Bungarus walli*	Wall’s krait	Northeast and Southwest	No	NA	-
13		*Naja oxiana*	Central Asian cobra	North and Northwest	No	Polyvalent	Iran, Pakistan, Uzbekistan, Egypt
14		*Naja sagittifera*	Andaman cobra	Andaman Islands	No	NA	-
15		*Ophiophagus hannah*	King cobra	South, Northeast, Andaman Islands	No	Polyvalent and monovalent	Thailand
16	Viperidae	*Gloydius himalayanus*	Himalayan pit viper	North	No	NA	-
17		*Protobothrops jerdonii*	Jerdon’s pit viper	Northeast	No	NA	-
18		*Protobothrops kaulbacki*	Kaulback’s lance-headed pit viper	Northeast	No	NA	-
19		*Protobothrops mucrosquamatus*	Brown-spotted pit viper	Northeast	No	Monovalent antivenom	China
20		*Trimeresurus gramineus*	Common bamboo pit viper	South and East	No	NA	-
21		*Craspedocephalus malabaricus*	Malabarian pit viper	Southwest	No	NA	-
22		*Macrovipera lebetina*	Levantine viper	Northeast	No		
23		*Protobothrops himalayanus*	NA	North	No	NA	-
24		*Trimeresurus andersonii*	Andaman Pitviper	Andaman Islands	No	NA	-
25		*Trimeresurus erythrurus*	Bamboo pitviper	East	No	Monovalent	Thailand
26		*Trimeresurus gumprechti*	Gumprecht’s green pit viper	Northeast	No	NA	-
27		*Craspedocephalus macrolepis*	large-scaled pit viper	South	No	NA	-
28		*Trimeresurus salazar*	Salazar’s pit viper	Northeast	No	NA	-
29		*Trimeresurus septentrionalis*	Nepal pitviper	North	No	NA	-
30		*Craspedocephalus strigatus*	horseshoe pit viper	South	No	NA	-
31		*Trimeresurus yunnanensis*	Yunnan bamboo pitviper	North	No	NA	-

NA—Not available.

**Table 2:** List of antivenom manufacturers in India.

SI No.	Name	Antivenom Manufacturer	Listed in WHO Database	Nature of Antivenom	Stated Efficacy
1	Polyvalent Snake Antivenin	Biological E Limited (Telangana, Hyderabad)	Yes	Polyvalent (raised against big four Indian snakes)	After reconstitution, each mL of Polyvalent snake venom antiserum neutralizes not less than: Indian Cobra venom—0.60 mg Common Krait venom—0.45 mg Russell’s viper venom—0.60 mg Saw scaled viper venom—0.45 mg (The stated efficacies of each of these antivenoms are indicated based on the LD_50_ and ED_50_ values obtained after performing In vivo studies)
2	Snake Venom Antiserum I.P.	VINS Bioproducts Ltd. (Telangana, Hyderabad)	Yes		
3	Polyvalent Snake Antivenom	Bharat Serums & Vaccines (Mumbai, Maharashtra)	Yes		
4	Snake antivenin I.P.	Haffkine Biopharmaceutical Corporation Ltd. (Mumbai, Maharashtra)	Yes		
5	Virchow (V-ASV)	Virchow biotech private limited (Telangana, Hyderabad)	No		
6	Snake Venom Antiserum I.P.	Premium Serums & Vaccines Pvt. Ltd. (Pune, Maharashtra)	No		
7	Snake Venom Antiserum I.P.	Mediclone Biotech (Chennai, Tamil Nadu)	No		
8	Polyvalent Anti Snake Venom Serum I.P.	King Institute of Preventative Medicine and Research (Chennai, Tamil Nadu)	Yes		

**Table 4:** Studies on using aptamers to treat snakebite envenomation.

SI No.	Species	Protein	Protein Family	Nature of Aptamer	Methodology Used for Selecting Aptamer	Efficacy/Efficiency	Reference
1	*Daboia russelii*	Daboxin P	Phospholipase A_2_	Nucleic acid aptamer	Entropy fragment-based approach and seed and grow method	Showed PLA_2_ inhibitory and anticoagulant activities.	[101]
2	*Bungarus multicinctus*	β-bungarotoxin (β-BuTx)	Neurotoxin (three-finger toxin)	DNA aptamer	plate-SELEX	The designed aptamer βB-1 was specific to β-BuTx and could differentiate *B. multicinctus* venom among the other snake venoms tested.	[99]
3	*Bungarus caeruleus*	α-Toxin	Neurotoxin (three-finger toxin)	Truncated aptamer	Truncated aptamer	A truncated DNA aptamer, α-Tox-T2, generated to fight against the α-Toxin of *Bungarus multicinctus* was also able to detect *Bungarus caeruleus* venom.	[96]
4	*Bungarus multicinctus*	α-bungarotoxin	Neurotoxin (three-finger toxin)	DNA aptamer	Single-step selection on a glass coverslip using designed aptamers	Simple one-step selection could be applied for the rapid production of DNA and RNA aptamers.	[90]
5	*N. atra*	Cardiotoxins	Neurotoxin (three-finger toxin)	DNA aptamer	Neogene Biomedicals Corporation synthesized the labeled single-stranded DNA samples	The aptamers designed to fight against *Bungarus multicinctus* α-bungarotoxin inhibited cytotoxicity and membrane damage induced by *Naja atra* cardiotoxins.	[95]
6	*Bungarus caeruleus*	β-Bungarotoxin	Neurotoxin (three-finger toxin)	DNA aptamer	SELEX	The designed aptamer could discriminate *B. caeruleus* venom from Russell’s, Cobra, and Saw-scaled viper’s venom and was specific to β-Bungarotoxin.	[100]
7	*C. rhodostoma* and *B. atrox*	Ancrod and batroxobin	Snake venom serine protease	ssDNA aptamers	SELEX	The toxin-specific aptamers were found to exhibit in vitro cross-reactivity against the different isoforms present in various snake species.	[93]

**Table 5:** Studies on developing alternative treatment strategies using phage display.

SI No.	Snake Species	Neutralizing Toxin/Protein (Antigen)	Phage Bound Molecule	Study Design	Reference
1	*Naja kaouthia*	α−cobratoxin	8-mer peptide	In vitro	[120]
2	*Naja naja atra*	*Naja naja atra* proteins (NNA proteins)	Single-chain variable fragment (scFv)	In vitro and in vivo	[121]
3	*Trimeresurus stejnegeri*	Whole venom	scFv	In vitro and in vivo	[122]
4	*Naja. nigricollis*, *Naja. mossambica*, and *Naja. melanoleuca*	Whole venom	scFv	In vitro	[123]
5	*Naja kaouthia*	Whole venom	Monoclonal antibody	In vitro and in vivo	[124]
6	*Bothrops jararacussu* and *Crotalus durissus terrificus*	Whole venom	scFv	In vitro and in vivo	[125]

**Table 7:** Studies on natural product-based inhibitors against snake venom.

SI No.	Species	Plant Extract/Compound	Protein Family	Nature of Plant Product	Neutralization Studies	Reference
1	Russell’s viper	Aristolochic acid	Phospholipase A_2_ L-amino acid Oxidase	Nitrophenanthrene carboxylic acids	In vitro cell based assays and in vivo mouse model	[101]
2	*Daboia russelli*, *Naja naja*, *Naja kaouthia*	Leaf extract of *Azadirachta indica*	Phospholipase A_2_	Non- terpenoids	In vitro assays and in vivo mouse model	[99]
3	*Daboia russelli*, *Naja naja*, *Echis carinatus*	Root extract of *Mimosa pudica*	Hyaluronidase and Protease	Not determined	In vitro assays	[96]
4	*Daboia russelli*	Leaf extract of *Morus alba*	Snake venom metalloproteinase and hyaluronidase	Not determined	In vitro assays and in vivo mouse model	[90]
5	*Naja naja*	Quercetin-3-O-α-rhamnoside	Phospholipase A_2,_ hyaluronidase and hemolytic activity	Flavonoid glycoside	In vitro assays and in vivo mouse model	[95]
6	*Naja kaouthia*, *Ophiophagus hannah*, *Daboia russelii*, and *Echis carinatus*	2-hydroxy-4-methoxy benzoic acid	Phospholipase A_2,_ snake venom metalloproteinase	Salicylates derivative	In vitro assays and in vivo mouse model	[100]
7	*Naja naja*	*Withania sominifera* Glycoprotein	Phospholipase A_2_	Glycoprotein	In vitro assays and in vivo mouse model	[93]

## Data Availability

Not applicable.
